# Accurate Receptor-Ligand Binding Free Energies from Fast QM Conformational Chemical Space Sampling

**DOI:** 10.3390/ijms22063078

**Published:** 2021-03-17

**Authors:** Esra Boz, Matthias Stein

**Affiliations:** Max Planck Institute for Dynamics of Complex Technical Systems, Molecular Simulations and Design Group, 39106 Magdeburg, Germany; boz@mpi-magdeburg.mpg.de

**Keywords:** ligand binding free energy, DFT, conformational sampling, ligand-receptor binding, GFN2-xTB, conformational entropy

## Abstract

Small molecule receptor-binding is dominated by weak, non-covalent interactions such as van-der-Waals hydrogen bonding or electrostatics. Calculating these non-covalent ligand-receptor interactions is a challenge to computational means in terms of accuracy and efficacy since the ligand may bind in a number of thermally accessible conformations. The conformational rotamer ensemble sampling tool (CREST) uses an iterative scheme to efficiently sample the conformational space and calculates energies using the semi-empirical ‘Geometry, Frequency, Noncovalent, eXtended Tight Binding’ (GFN2-xTB) method. This combined approach is applied to blind predictions of the modes and free energies of binding for a set of 10 drug molecule ligands to the cucurbit[n]urils CB[8] receptor from the recent ‘Statistical Assessment of the Modeling of Proteins and Ligands’ (SAMPL) challenge including morphine, hydromorphine, cocaine, fentanyl, and ketamine. For each system, the conformational space was sufficiently sampled for the free ligand and the ligand-receptor complexes using the quantum chemical Hamiltonian. A multitude of structures makes up the final conformer-rotamer ensemble, for which then free energies of binding are calculated. For those large and complex molecules, the results are in good agreement with experimental values with a mean error of 3 kcal/mol. The GFN2-xTB energies of binding are validated by advanced density functional theory calculations and found to be in good agreement. The efficacy of the automated QM sampling workflow allows the extension towards other complex molecular interaction scenarios.

## 1. Introduction

Binding free energy calculations on molecular systems are required to eventually predict binding affinities for (bio)molecular systems [1]. Especially during the last decade, the pharmaceutical industry and further research activities along this line are gaining continuous attention in order to obtain more efficient and selective drugs [2,3,4].

The use of computational drug design tools enable to reduce the traditional and time-consuming lab experiments in lead optimization [5]. These tools are also crucial to understand the underlying mechanisms of drug binding and metabolism. However, there are many factors in molecular recognition, which contribute to the accuracy of a calculation such as the sufficient sampling of the conformational space, energy evaluation schemes, maybe scoring functions for binding energy calculations, and the treatment of solvation. For example, enantioselective receptor binding of chiral compounds required a re-parametrization of atomic charges and torsional profiles, an exhaustive conformational Monte Carlo sampling plus a systematic low-mode conformational search followed by a normal mode integration approach to obtain enantiomeric excess in good agreement with experiments [6,7,8].

The number of computational approaches to calculate Gibbs free energies of binding is large and increasing: All-atom molecular dynamics (MD) simulation with explicit solvent molecules plus efficient free-energy sampling tools are, in principle, able to accurately predict binding free energies of ligands to proteins [9]. Free-energy simulation approaches are complex and include the double-decoupling method (DDM) and potential of mean force approach (PMF) augmented by either free-energy perturbation (FEP), thermodynamic integration (TI), or umbrella sampling. There are a number of comprehensive review articles, which address various aspects of free energy calculations [9,10,11,12].

**Figure 1:**
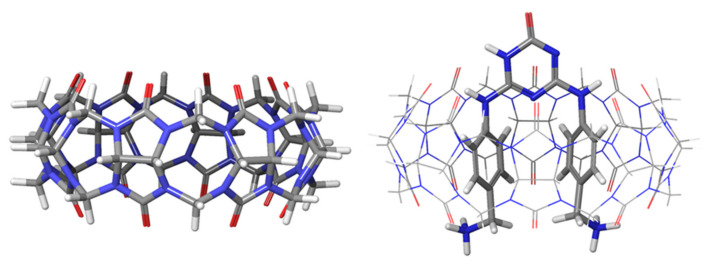
Structure of the host molecule CB[8] (side view) and co-crystallized with G0 ligand.

In order to assess the performance of those approaches, Statistical Assessment of Modeling of Proteins and Ligands (SAMPL) blind prediction challenges were initiated to address various aspects of predictions of properties related to drug discovery [13,14,15,16]. Since 2008, those SAMPL competitions have addressed the accurate modeling of protein-ligand complexes, but also small molecule properties such as hydration energies and the binding thermodynamics of host-guest systems. The most recent SAMPL8 was calling for predictions of ‘drugs of abuse’ binding to a synthetic receptor, including morphine, hydromorphine, methamphetamine, cocaine, and others. Cucurbit[n]urils, CB[n], are macrocyclic synthetic receptors and constructed by glycoluril groups, which are connected by [n] units of methylene bridges [17]. Due to their high binding constants and non-toxic nature, they are very good candidates for biological applications [18]. CB[8] is the largest member of this family. It has a cavity diameter of 8.8 Å and a cavity volume of 479 Å^3^ (see Figure 1) [17]. These features make CB[8] a very good candidate not only for 1:1, but also for possibly 1:2 host-guest complexation [19]. Binding constants K_a_ in the range of 10^4^–10^12^ M^−1^ of several CB[n] complexes (with CB[6], CB[7] and CB[8] as hosts) were measured by monitoring ^1^H-NMR spectra at neutral pH [20].

Cucurbit[n]urils were already included in previous challenges [14,16,21]. Several methods like MD simulations, meta-dynamic simulations, clustering, docking, umbrella sampling, QM, and QM/MM approaches were used to treat the given systems of the challenge [22,23,24,25,26,27]. Analysis of the submitted results revealed that the accuracy of force fields, inadequate consideration of different protonation states, and protomers of the structures were decisive factors for deviations from experiment [14].

Association free energies for supramolecular host-guest complexes can be computed with good accuracy by dispersion corrected density functional theory (DFT-D3) using a large basis set and one or a few manually generated possible binding modes [28,29].

Generally, for the modelling of proteins and biologically relevant large structures, classical MD simulations are preferred due to their low computational cost. These methods are usually lacking the required accuracy since even the most specialized force fields are missing some important parameters to describe the electronic properties of a system. Gibbs free energies of binding (ΔG_binding_) are computationally demanding since exhaustive conformational sampling plus the accuracy of interaction energies are both essential.

Here, we present results of the automated exploration of the chemical space using CREST (Conformer-Rotamer Ensemble Sampling Tool), which makes use of the fast semi-empirical QM tight-binding ‘Geometry, Frequency, Noncovalent, eXtended Tight Binding’ GFN2-xTB method [30]. GFN2-xTB is a new and extended semi-empirical tight-binding model, which is especially parametrized for geometries, frequencies, and non-covalent molecular interaction energies. A minimal valence basis set of atom-centered Gaussian functions is employed. Due to the inclusion of short-range dispersion interactions, the results are less empirical, and the method is more physically sound compared to other semi-empirical approaches. In contrast to previous semi-empirical methods, which rely on an element pair-specific parametrization, GFN2-xTB strictly follows a global and element-specific parameter strategy in which no element pair-specific parameters are employed.

The CREST workflow makes use of iterative metadynamics plus a genetic Z-matrix crossing (iMTD-GC) approach to automatically sample the low-energy conformational space of ligands, receptors, and ligand-receptor complexes at finite temperature. For an accurate calculation of the Gibbs free energies of binding, it is necessary to include all thermally accessible molecular conformations by Boltzmann weighting of the conformer-rotamer ensemble (CRE). Conformers and rotamers are discriminated by a combination of structural (RMSD and rotational constants) and energetic threshold criteria (ΔE).

Hybrid and double-hybrid DFT calculation were used to verify the ligand poses and their binding energies since those methods showed a low mean absolute deviation for the S22 benchmark set of non-covalent interactions [31].

This combinatorial workflow of an exhaustive conformational and rotational sampling from meta-dynamics with different bias potentials and normal MD sampling for low energy conformers gives a representative set of CREs. The GFN2 calculated Gibbs free energies of binding for the SAMPL8 ‘drugs of abuse’ compounds to the cucurbit[8]uril, CB[8] receptor are in excellent agreement with the recently released experimental data [32]. This demonstrates the efficiency of the CREST iterative meta-dynamics with genetic crossing (iMTD-GC) exploration of chemical space plus the accurate description of non-covalent free energies of ligand-receptor binding; it holds great promise for calculating absolute binding energies of small drug molecules in receptor binding sites.

## 2. Results and Discussion

**Figure 2:**
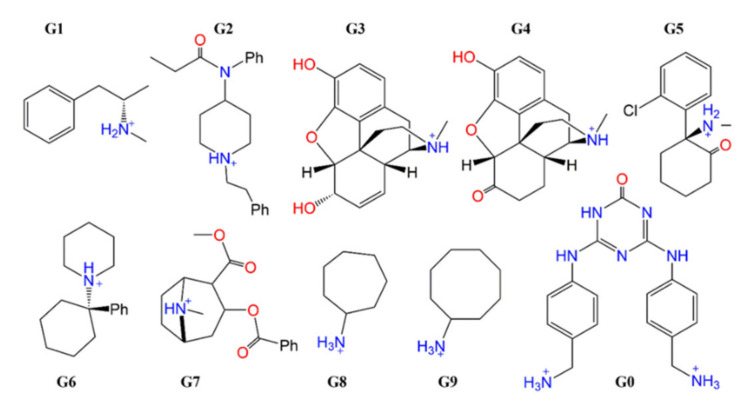
Structures of the guest molecules (**G0**–**G9**) used for calculations of Gibbs free energies of binding to the host CB[8].

The receptor cucurbit[8]uril (CB[8]) has a cage-like form (Figure 1) and can form complexes with different guest molecules. Ten complex ligands (Figure 2, **G0** to G**9**) are included in this work. For ligand **G0**, a CB[8]*G0 1:1 co-crystal structure is available together with experimental Gibbs free energies of binding [20]. This ligand-receptor complex thus serves as a benchmark to ensure that the GFN2 calculations are able to reproduce experimental structural parameters and CREST is able to recover experimental ligand binding free energies.

### 2.1. Structural Parameters of Host and Host-Guest Complexes

**Figure 3:**
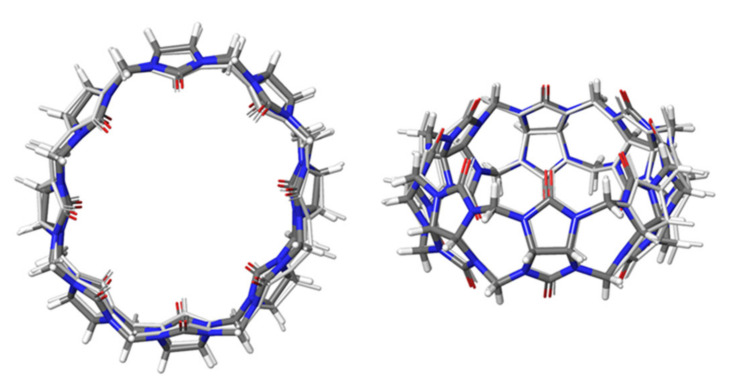
Structural superposition of the experimental X-ray structure of the CB[8] receptor (in color) and the GFN2-xTB-optimized structure. Left: Top view; Right: Side view.

In the ligand-receptor complex CB[8]*G0, the receptor has a centrosymmetric arrangement whereas the diamine ligand slightly deviates from symmetric binding. In addition, the diamine is disordered inside the CB[8] crystal structure in a 2:1 ration. Co-crystallized iodine (I_2_) and most of the water molecules do not occupy symmetry-related positions. In the workflow of calculating accurate Gibbs free energies of binding, the generation of reliable poses is the first step. Figure 3 shows a comparison of the GFN2-xTB optimized structure of CB[8] with the crystal structure. The root mean square deviation (RMSD) of the xTB-optimized structure and the experimental one is 0.3 Å. This shows (i) the accuracy of the chosen computational method, and (ii) the fact that the CB[8] ring does not deform upon ligand binding.

The crystal structure of CB[8] in complex with the **G0** ligand in a 1:1 stoichiometry [20] was first energy minimized. The RMSD between the CB[8]*G0 host-guest co-crystal and the GFN2-xTB optimized structures was 0.50 Å. In particular, the re-adjustment of the orientation of the protonated amines to form two hydrogen bonds with the carbonyl oxygen atoms led to a RMSD of 0.36 Å for the **G0** ligand only (see below).

We carefully assessed the degree of flexibility of the CB[8] receptor and its effect on the sampling of the Gibbs free energy. CREST searches of the receptor structure with and without constraining the host atoms gave identical results. Six iterative MTD runs with different bias potentials, a re-optimization of the generated conformers plus a re-ranking according to calculated Gibbs energies gave only two representative CB[8] conformers, in the presence and absence of positional constraints. The lowest conformer has a population of 99.96% and corresponds to the crystallized receptor structure. The Gibbs free energy difference between a constrained and flexible host molecule was −0.5 kcal/mol. Thus, the CB[8] receptor can be constrained as one entity using a force constant of 0.5 without any loss in accuracy.

**Figure 4:**
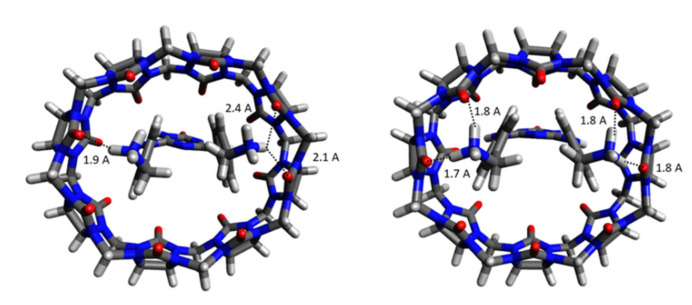
Ligand **G0** inside the CB[8] receptor. Left: X-ray structure of co-crystallized complex. Right: Top-ranked ligand-receptor complex from conformational searches and ranking according to Gibbs free energies.

Then, ligand and receptor-ligand complex were subjected to individual exhaustive CREST searches. The **G0** ligand adopts a U-shape conformation inside CB[8]. A comparison of the co-crystallized ligand **G0** inside the CB[8] receptor and the best-ranked pose according to its Gibbs free energy of the receptor-ligand complex is given in Figure 4. The small difference in Gibbs free energy of binding of **G0** to the CB[8] host is 0.6 kcal/mol and shows that constraining the host molecule during CREST ligand-receptor sampling is possible without a loss in accuracy.

### 2.2. The Protonation State of G0

The melamine-derived ligand **G0** (1,3-bis(4-aminomethylphenyl)triazene) only slightly deforms the host in the CB[8]*G0 complex (see above). The free ligand **G0** shows 12 different conformers and rotamers but complexation induces a folding process resulting in the U-shaped structure of **G0** in the CB[8]*G0 complex with 8 members in the CRE. The X-ray crystal structure of host-guest complex demonstrated the excellent fit of the U-shaped conformer and the cavity of CB[8] [20]. The CB[8]*G0 complex exhibits an unusually slow dissociation with a half-life of ~1 day, which was attributed to a large conformation re-arrangement of the ligand before dissociation. The room temperature ^1^H-NMR spectrum in D_2_O was used to assign a doubly protonated states to the 4-aminomethyphenyl groups. When calculating the ligand binding free energy of the doubly protonated ligand **G0^2+^** to CB[8], a value of −35.93 kcal/mol was obtained for the top-ranked pose and −37.30 kcal/mol for the Gibbs free energy of binding for the ensemble. These values are more than a factor of two larger than experiment (−14.67 kcal/mol) and larger than that observed for all other host-guest complexes (see below). DFT single point calculations for the top-ranked binding pose gave consistent binding energies of −40.19 kcal/mol (PWPB95/def2-TZVP) and −45.67 kcal/mol (B2PLYP/def2-TZVP), which are in good agreement with the GFN2-xTB results but also significantly larger than the experiment.

We then investigated the binding of a neutral **G0^0^** to CB[8] for which a Gibbs free energy of binding of −4.06 kcal/mol was obtained, which is too low and not in agreement with the experiment. Only for the singly protonated **G0^+^**, reasonable ligand binding free energies of −18.25 kcal/mol (top-ranked pose) and −20.02 kcal/mol (ensemble) were obtained. The energies of association (ΔE_a_) for the best-ranked binding mode was −38.61 kcal/mol for GFN2-XTB; DFT calculations with PBE0 (−31.12 kcal/mol), B2PLYP (−37.68 kcal/mol) and PWPB95 (−33.77 kcal/mol) give a very consistent view and support the assignment to a mono-protonated state of **G0**. Thus, we can assign a singly protonated form to **G0**.

The assignment of a doubly protonated form to **G0** was based on the interpretation of ligand ^1^H-NMR spectra [20]. In D_2_O, the amine protons are exchanging to deuterons and not detectable in the spectrum. The equivalence of chemical shifts of the aromatic hydrogen atoms of the phenyl rings was used to suggest identical protonation states of the 4-aminomethyl groups. However, a fast proton exchange at room temperature also renders them apparently equivalent. The experimental and calculated free energies of binding are very close to those of the other ligands in this competition and allows to assign a mono-protonated state.

### 2.3. Gibbs Free Energies of Ligand-Receptor Binding

Accurately calculating the Gibbs free energies of ligand-receptor binding requires an exhaustive conformational search plus a reliable energy evaluation and Boltzmann weighting.

Table 1 gives the total number of unique structure in the CREs after conformational sampling, elimination of duplicate entries using structural and energetic criteria, followed by a re-optimization with tight convergence criteria and prior to thermodynamic corrections from Hessian calculations. The numbers of unique structures are thus corresponding to the number of free energy calculations, which need to be performed to obtain free energies of binding. It is evident that an accurate but also very efficient method is required to calculate Gibbs free energies of 136 CB[8]*G2 receptor-ligand complexes, for example.

The number of unique entries after the first two stages can be found in the Supplementary Information Table S2.

### 2.4. Free Energies of Binding of Cyclic Anamines to CB[8]

The binding of ligands **G8** (cycloheptanamine) and **G9** (cyclooctanamine) to CB[8] is a benchmark for the sufficiency of our conformational sampling, and the accuracy of ensemble-averaged Gibbs free energies of interactions (see Figure 2). As part of SAMPL6 [14] and later available experimental ligand binding affinities, they serve as competitive inhibitors for ligand binding in NMR titration experiments. As a result of CREST sampling, CREs with 90 and 175 unique entries for the ligand-receptor complexes, and 17 and 22 unique free ligand structures were generated after sequential refinement (see Table 1). This demonstrates that, albeit a low degree of free ligand conformational flexibility, the receptor-ligand complexes display a very complex potential energy surface and a multitude of distinct binding events.

**Figure 5:**
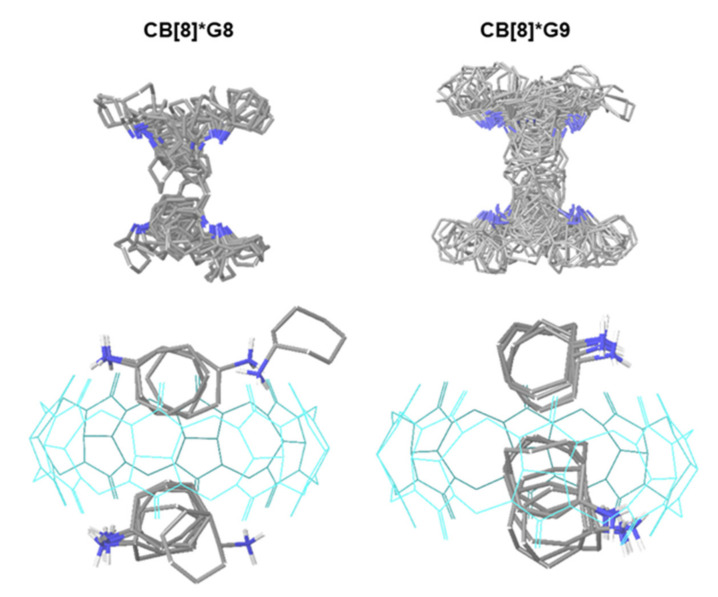
Top: Display of the 90 and 175 unique representatives of the CREs of the CB[8]*G8 and G9 complexes, respectively. The CB[8] receptor is not displayed. Bottom: Binding modes of the 10 top-ranked poses (CB[8] is displayed in cyan and wireframe for the sake of clarity).

Figure 5 displays an overlay of the unique entries in the ligand-receptor CREs. Due to the symmetry of the host, the **G8** and **G9** ligands can access the CB[8] receptor from above and below the cage. Both binding modes are fully recovered during the sampling. In the lower part of Figure 5, the top-ranked 10 binding poses of the ensembles are shown. This gives an indication of the extent of variability of ligand binding modes to the receptor in a narrow energy window of 6 kcal/mol. The alkyl ring of the ligands is mainly pointing towards the inner side of the receptor where weak hydrophobic interactions are dominating. The positively charged amine groups, on the other hand, are mostly directed towards the carbonyl groups of the CB[8] receptor to form hydrogen bonds.

The calculated free energies of binding for the **G8** and **G9** ligands are −8.30 and −8.64 kcal/mol when considering the top-ranked conformer only, compared to an ensemble ΔG_bind,ens_ of −7.90 and −8.13 kcal/mol, respectively (Table 2). From a large number of poses, ligand-receptor complexes with 90 and 175 unique conformer entries can efficiently be generated with our approach, sampled, and ranked. The incorporation of conformers above the global minimum affects the calculated ligand binding free energies to a small degree only. In absence of any structural information, the conformational sampling plus sequential refinement steps and final re-ranking according to their Gibbs free energies is able to generate ligand binding modes in excellent agreement with experiment: for **G8** and **G9**, the experimental binding energies of −9.18 and −10.2 kcal/mol are only 0.8–1.6 kcal/mol lower than the GFN2-xTB results.

**Figure 6:**
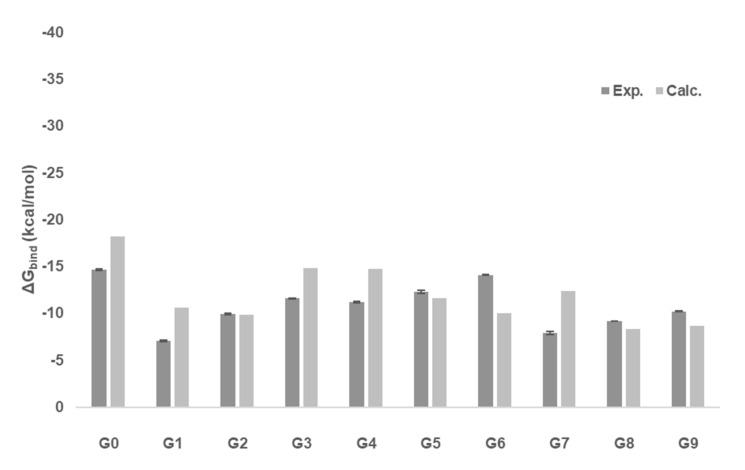
Calculated Gibbs free energies of binding of top-ranked ligands **G0**-**G9** (in kcal/mol) to the CB[8] receptor in comparison to experiment.

Figure 6 shows a comparison of the GFN2-xTB **G0**–**G9** calculated ligand binding free energies with experimental values, which were released after the SAMPL challenge. The calculated Gibbs free energies of binding (see Table 2) are in good agreement with the experiment. For one-half of the complexes, the GFN2-xTB free energies of binding are more negative than experiment. An overbinding by 3.2 and 3.5 kcal/mol is observed for ligands **G3** (morphine) and **G4** (hydromorphone) and by 4.5 kcal/mol for **G7** (cocaine). The only structural motif that they have in common is the tricyclo-bridged hydrocarbon with a positively charged tertiary amine incorporated in one of the rings. This appears to be a critical structural issue for the xTB calculations.

**Figure 7:**
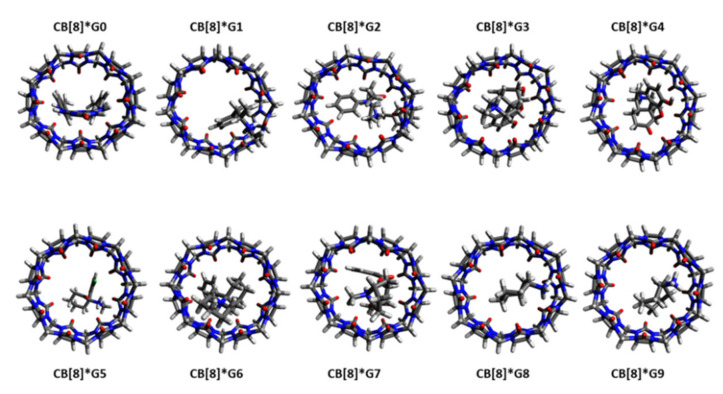
Structures of the top-ranked CB[8]*G0–G9 receptor-ligand complexes.

Figure 7 displays the top-ranked poses of ligands **G0** to **G9** binding to the curcurbit[8]uril receptor. For methamphetamine (G1) binding to CB[8], free energies of binding of −10.61 kcal/mol and −12.22 kcal/mol were calculated for the top-ranked binding mode and the ensemble, respectively. With respect to the experimental value of −7.05 kcal/mol, the typical overbinding can be observed. The molecule is small and there are 21 unique entries in the CRE of the ligand-receptor complex (see Table 1). The phenyl ring is incorporated into the hydrophobic cavity of the host molecule. The propane-2-amine is pointing towards the solvent and the amine group forms two hydrogen bonds with carbonyl groups of CB[8] receptor with distances of 1.8 Å and 1.9 Å.

Fentanyl (**G2**) has a calculated free energy of binding of −9.84 kcal/mol (−11.14 kcal/mol for the CRE ensemble structures). This is in excellent agreement with the experimental value of −9.94 kcal/mol. Given its large number of rotatable bonds (6), the free ligand has 158 unique conformers. Due to its elongated shape, 137 unique structures were also found for the ligand-receptor complex. The two terminal phenyl rings at each end of the complex, plus the central piperidine make this compound amphiphilic in character. The hydrophobic *N*-phenyl propylamide occupies a position inside the CB[8] ring, the piperidine forms hydrogen bonds with the ring atoms at 1.8 Å distance, and the (hydrophobic) ethylphenyl moiety is solvent-exposed.

The binding of morphine (**G3**) to the host has an experimental Gibbs free energy of −11.6 kcal/mol which agrees well with the calculated values of −14.80 kcal/mol and −16.31 kcal/mol. The morphine molecule is rigid and has no freely rotatable bonds. This explains why only four unique structures of morphine poses of binding in the complex are found. Whereas the 3-methyl-hexahydro isoquinoline is solvent-exposed and does not form direct interactions with the host, one diol forms a weak H-bond of 2.1 Å distance with a carbonyl oxygen atom of the receptor. There are no further directed intermolecular interactions.

The experimental hydromorphine (**G4**) binding free energy (−11.2 kcal/mol) is close to the calculated (−14.74 and −16.13 kcal/mol). The xTB results may even reproduce the relative binding affinity differences between **G4** and **G3**. In addition, the ligand-receptor interactions are similar. There is only one H-bond between ligand and receptor at a distance of 2.1 Å.

The ligands **G5** (ketamine) and **G6** (phencyclidine) are similar in shape and possess 10 and 19 unique structures, respectively, in the final CRE. Ligand **G5** (ketamine) does not fully insert into the CB[8] cavity. The top pose is positioned above the receptor: The phenyl ring is in an apical position with the chloride pointing towards the receptor’s cage. The cyclohexanone ring occupies an equatorial position. Ketamine only forms one strong hydrogen bond between the amine and the carbonyl group of the receptor (distance 1.7 Å). Given the excellent agreement between calculated and experimental ligand binding free energies, we are confident to assign a +1 total charge to the system although experiments were performed at close to neutral pH. The best-ranked phenylcyclidine ligand (**G6**) is, in contrast, buried in the receptor with both cylcohexane and phenyl rings inserted. The positively charged piperidine is in an apical position and pointing towards the solvent. It forms a hydrogen bond at 1.8 Å distance with the receptor. The calculated free energies of binding (−11.62 kcal/mol for top pose and −12.95 kcal/mol for the ensemble of **G5**) vs. −10.03 kcal/mol and −11.30 kcal/mol for **G6** are in good agreement with the respective experimental values of −12.3 and −14.1 kcal/mol.

In the receptor-ligand complex CRE of cocaine (ligand **G7**) 39 unique structures were found. The deviation between experiment (−7.93 kcal/mol) and XTB calculations (−12.38 kcal/mol for top pose and −13.54 kcal/mol for the ensemble population) is 4.5–5.5 kcal/mol and thus the largest of the set. The azabicyclooctane is located above the receptor; the benzoyloxy and methyl carboxylate groups are fully incorporated into the receptor. There is no directed interaction between ligand and receptor.

**Figure 8:**
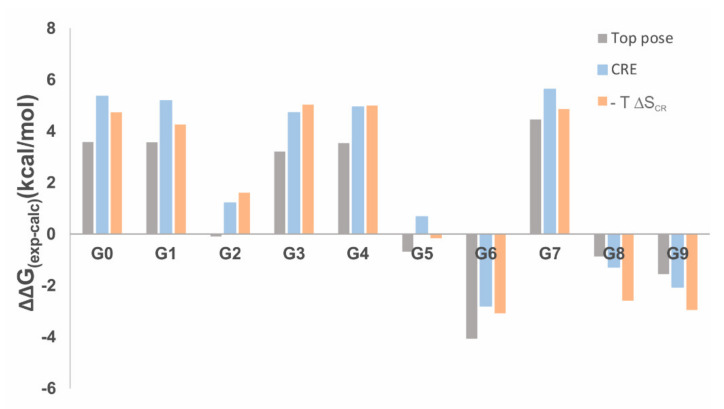
Deviations of GFN2-xTB CREST results from experiment in kcal/mol for ligands **G0** to **G9** binding to CB[8]. Grey: Gibbs free energy of binding for top pose only; blue: For all members of the CRE; orange: Taking into account changes in conformational and rotational entropy of the ensemble (−TΔS_CR_).

Figure 8 gives the deviation of xTB calculated Gibbs free energies of binding from experiment (see Table 2). A positive value refers to an overbinding of GFN2-xTB calculations. In 7 out of the 10 complexes, this overbinding is apparent. When only the top pose is considered, the mean absolute error (MAE) is 2.6 kcal/mol, which increases to 3.38 kcal/mol when including the Boltzmann-weighted ensemble population (see Table 3). When the change in conformational and rotational entropy is considered (−T ΔS_CR_), the error is 3.43 kcal/mol.

### 2.5. Comparison of GFN2-xTB Results with DFT

The GFN2-xTB Hamiltonian is especially parametrized for properties such as geometries, vibrational frequencies, and non-covalent interactions [34]. Usually, covalent bond energies are systematically overestimated by this method. For non-covalent interactions, however, GFN2-xTB outperforms GFN-xTB and PBE0-D3(BJ) without specific terms in the Hamiltonian to treat hydrogen bonds.

Table 4 compares the *energies of association* (E_a_) from GFN2-xTB with DFT calculations using hybrid PBE0, double-hybrid B2PLYP and the spin-opposite-scaled double hybrid functional PWPB95 of ligands **G0**–**G9** with the CB[8] receptor. GFN2-xTB shows the abovementioned systematic overbinding and the calculated energies of association are larger by up to 25 kcal/mol relative to DFT calculations.

All DFT results give a very consistent picture of the energies of association of ligands **G0**–**G9** with the receptor. Hybrid, double-hybrid, and spin-opposite-scaled double hybrid functionals agree to within 4–7 kcal/mol. This is a strong indicator of the reliability and accuracy of the GFN2 generated best binding poses. The generated binding modes are very well suited for any subsequent higher-level calculation.

The DFT energies of association are consistently lower than the GFN2-xTB results. B2PLYP calculations give larger E_a_ (by a mean of −4 kcal/mol) than PBE0; and PWPB95 (with a mean deviation of −1.4 kcal/mol from PBE0 results) is in between. However, since GFN2 is particularly parametrized for the thermochemistry of non-covalent interactions, a comparison between the GFN2 Gibbs free energies and DFT electronic energies appears more plausible.

## 3. Materials and Methods

### 3.1. Host-Guest Starting Structures

The initial structures of the individual host and guest molecules (Figure 2) were taken from the from the SAMPL8 repository (https://zenodo.org/record/4029560; accessed on 13 March 2021) [35]. The host molecule cucurbit[8]uril (CB[8]) is a cyclic neutral structure with eight carboxylic acid groups. Its cage-like form enables the formation of non-covalent receptor-ligand complexes with a diverse set of ligands. This cavity has a hydrophobic binding pocket, which eases the binding upon weak hydrophobic interactions. We took the CB[8] crystal structure from ref [20] as the receptor structure.

After carefully checking the effect of the host flexibility, we constrain the CB[8] during the simulations.

Due to the pK_a_ value of the ligands’ amine groups and the given experimental conditions of SAMPL8 challenge (pH = 7.4 at 25 °C), all the ligands were submitted to the calculations in their protonated states. The ligands **G1**–**G9** (Figure 2) are +1 charged, whereas the co-crystallized **G0** ligand has a charge of +2 [20]. A GBSA solvent model for water used and there were no counterions present. Experimental conditions were as follows: The buffer was 20 mM sodium phosphate buffer at pH 7.4. Guest concentrations were in the 0.5 to 1.5 mM range and CB8 concentrations between 0.025 to 0.1 mM. All binding stoichiometries have been validated by repeat ITC experiments and by NMR spectroscopy binding studies. Ligands **G0**–**G9** were created with Maestro and stored as Cartesian coordinations.

They were manually positioned inside the CB[8] receptor structure. These are the co-crystallized ligand 1,3-bis(4-aminomethylphenly)triazene (**G0**), methamphetamine hydrochloride (**G1**), fentanyl citrate (**G2**), morphine hydrochloride (**G3**), hydromorphine hydrochloride (**G4**), ketamine hydrochloride (**G5**), phencyclidine hydrochloride (**G6**), cocaine hydrochloride (**G7**), cycloheptanamine hydrochloride (**G8**), and cyclooctanamine hydrochloride (**G9**). We also invested the possibility of chiral discrimination by the receptor; for example, the difference in Gibbs free energy of binding of R- and S-enantiomers of **G5** to CB[8] was below 1 kcal/mol and not further considered.

### 3.2. Structural Optimization and Free Energies of Binding

For the geometry optimizations and sampling of the host-ligand complexes, we have used CREST [30,36] (Conformer-Rotamer Ensemble Sampling Tool, v2.10.2; available from https://github.com/grimme-lab/crest, accessed on 13 March 2021) and GFN2-xTB (v6.3.2; available from https://github.com/grimme-lab/xtb, accessed on 13 March 2021) [37,38].

Due to the large number of atoms in biologically relevant structures and the flexibility of either receptor and/or ligands, full QM calculations are time-consuming and impractical for most application to these systems. XTB is able to perform very fast and accurate semi-empirical quantum mechanical calculations [39] including geometry optimizations for complexes of 200–300 atoms, vibrational frequencies, and thermodynamic corrections in the rigid-rotor harmonic oscillator (RRHO) approximation [34]. The Generalized Born (GB) model augmented with the hydrophobic solvent accessible surface area (SA) term (GBSA) was used to model solvent effects.

Initial binding poses of the host-guest systems were generated manually by positioning the ligand close to the center of the receptor. Then, the geometries were refined in order to remove steric clashes.

The free energy of binding of the top-ranked pose is calculated at T = 298.15 K in aqueous solution according to Equation (1):

(1) $$\Delta G_{binding}=\Delta E_{\left(complex-receptor-ligand\right)}\;+\;\Delta G_{RRHO}^{T}\;+\;\Delta G_{GBSA}$$

The Gibbs free energy of the ensemble ΔG_bind,ens_ is the Boltzmann-weighted sum of all entries within an energy window of 6 kcal/mol above the global minimum. The conformational entropy of an ensemble is the entropy associated with the number of conformations of the molecule in the energy window. To calculate the conformational entropy, the possible conformations of the molecule are discretized into a finite number of states according to the CRE criteria. It is then calculated as the probability-weighted occupancy of each structure in the ensemble as S_CR_ = −R Σ_i_ p_i_ log p_i_, where R is the gas constant and p_i_ is the probability of the molecule to be in state _i_.

The ensemble entropy is also linked to the ensemble free energy G_CR_ = −T·S_CR_. It is common for a free ligand to lose conformational entropy upon forming a ligand-receptor complex. Here, sometimes the ligand in the receptor shows more possible binding modes than the free ligand has accessible conformers in solution.

### 3.3. The Conformer/Rotamer Ensemble

The exploitation of the conformational space by conventional sampling methods is limited. Here, meta-dynamic molecular dynamics simulations (MTD) are a superior tool for an extensive sampling of states and extraction of individual conformers. It establishes more reliable ensembles at a good accuracy/computational cost ratio [40]. The iMTD-GC procedure in CREST is a combined approach of extensive meta-dynamic sampling (MTD), with a final additional genetic Z-matrix crossing (GC) step in order to give a final conformer/rotamer ensemble (CRE). For complexes of CB[8] and **G0** to **G9**, the non-covalent interaction (NCI) mode creates an additional polynomial ellipsoid wall potential, which is added to the meta-dynamics. This avoids the dissociation of weakly bound aggregates.

XTB calculations are combined with root-mean-square-deviation (RMSD) based meta-dynamics by applying a Gaussian-type biasing potential to previous minima on the PES (Equation (2)):

(2) $$V_{bias}\;=\;\overset{n}{\underset{i}{\sum }}k_{i}\;\exp\left(-\alpha \Delta_{i}^{2\;}\right)$$

where Δ_i_ are collective variables, *n* is the number of reference structures, *k_i_* are the pushing strengths, and α determines the potential shape. The MTD simulation length is determined automatically by a flexibility measure of the molecule. Several independent MTDs (at 300 K) are performed with different settings for *α* (in Bohr^−1^) and *k_i_*/*N* (in m*E*h) (see Table S1)

The default settings are employed for the iMTD-GC procedure in CREST. The MTD simulation length is determined automatically by a flexibility measure of the molecule and varying settings for α and *ki*/*N*. Snapshots are geometry optimized in a multi-level, three-step-filtering procedure by applying two loose threshold settings. Short regular MD simulations are performed on the 6 lowest conformers at 400 and 500 K and followed by very tightly converged optimization and energy windows of 15, 10, and 6 kcal/mol.

The resulting ensembles were further refined and re-ranked using tighter optimization criteria. All structures were filtered with respect to their root-mean-square-deviation (RMSD), rotational constant (Be) and relative energies within 6.00 kcal/mol of the global minimum. Energy and matching structural criteria were employed to discard duplicates. In the next step, six iterative MD sampling runs were performed starting from the lowest energy conformers. All unique conformers and rotamers within the 6 kcal/mol energy window were assembled into a conformer/rotamer ensemble (CRE). Entries in the CREs were used for further optimization with tighter optimization criteria to generate the final ensemble of unique structures.

The NCI wall potential is removed for geometry optimizations. For each member of the final ensemble, structural optimizations with very tight convergence criteria and then vibrational frequencies were calculated in order to obtain thermodynamic corrections and Gibbs free energies. Finally, conformers in these ensembles were re-ranked according to their Gibbs free energies. Top-ranked poses of each ensemble were submitted further to higher level DFT calculations.

### 3.4. DFT Calculations

Hybrid DFT PBE0-D3 [41,42], double-hybrid B2PLYP [43], and double-hybrid-meta-GGA PWPB95 [44] were performed using ORCA v4.2.1 [45,46] in order to benchmark the GFN2-xTB energies of association. For these single point calculations, a def2-TZVP basis set with corresponding auxiliary basis sets and the RIJCOSX approximation were employed in order to reduce the computational cost [47,48,49]. Weak non-covalent interactions were considered by using D3 dispersion corrections with Becke–Johnson damping [29,50]. Solvation effects were considered using a CPCM implicit solvent model for water [51]. Only for **G7**, a structural re-optimization at the TPSS-D3(BJ)/def2TZVP level was performed.

## 4. Conclusions

The blind prediction of accurate ligand-receptor poses and free energies of binding shows that the efficient GFN2-xTB method can be combined with conformational sampling using meta-MD and normal MD in CREST. An elimination of duplicates in terms of structural criteria (RMSD and rotational constants) and energetics yields the conformation-rotamer ensemble CRE. With subsequent re-ranking steps using tighter criteria, finally the calculated free energies of binding of either the top poses only or the entire CRE are in good agreement with experiment.

The mean absolute error is comparable to that of usual predictions in the SAMPL host-guest competitions. Most of the entries, however, rely on a pre-parametrization of non-polarizable atomic partial charges from QM calculations. CREST with GFN2-xTB directly enters at the sampling stage without the need of a parametrization. It is also not relying on a priori knowledge of possible ligand binding modes.

For related CB[7] complexes, meta-GGA PW6B95 single point calculations with a COSMO-RS solvation model at TPSS-D3(COSMO) optimized structures gave a MAD of 2.02 ± 0.46 kcal/mol but energies were reported relative to the experimental free binding energy of CB[7]*1 [29]. Single-structure, non-dynamic models were generated by hand and selected candidate poses were subsequently optimized.

In this work, a comparable accuracy can be obtained without any manual intervention and experimental reference data. Due to the impressive computational speed and efficacy, CREST can also be applied to ligand-protein complexes. The ligand conformations in a drug-binding site can efficiently be sampled and provide reliable free energies of interactions. Such an approach will be of relevance also for covalent ligand binding, non-standard states of protonation, or post-translational modifications of either the ligand or receptor.

## Figures and Tables

**Table 1:** Number of unique structures in the conformer/rotamer ensembles (CREs) of free ligands **G0**–**G9** and ligand-receptor complexes CB[8]*ligand prior to final re-ranking according to Gibbs free energies.

	Number of Unique Entries in CRE of Free Ligand	Number of Unique Entries in the CRE of the CB[8]*Ligand Complex
**G0**	9	12
**G1**	11	21
**G2**	158	137
**G3**	5	4
**G4**	4	8
**G5**	9	10
**G6**	14	19
**G7**	16	39
**G8**	17	90
**G9**	22	175

**Table 2:** Comparison of calculated GFN2-xTB Gibbs free energies of binding of ligands **G0**–**G9** (in kcal/mol) to the CB[8] receptor with experiment from ref [33].

Ligand	Experiment	ΔG_bind_	ΔG_bind,ens_
**G0**	−14.67 ± 0.1^a^	−18.25	−18.25
**G1**	−7.05 ± 0.1	−10.61	−12.22
**G2**	−9.94 ± 0.06	−9.84	−11.14
**G3**	−11.6 ± 0.04	−14.80	−16.31
**G4**	−11.2 ± 0.1	−14.74	−16.13
**G5**	−12.3 ± 0.16	−11.62	−12.95
**G6**	−14.1 ± 0.04	−10.03	−11.30
**G7**	−7.93 ± 0.15	−12.38	−13.54
**G8**	−9.18 ± 0.03	−8.30	−7.90
**G9**	−10.2 ± 0.04	−8.64	−8.13

^a^ from ref. [20]; Experimental uncertainties are given. ΔG_bind_ is the free energy of binding of the top-ranked pose. ΔG_bind,ens_ is the Boltzmann-weighted Gibbs free energy of binding of all CREs from Table 1.

**Table 3:** Error of GFN2-xTB CB[8]*(G0–G9) calculated ligand binding free energies in kcal/mol.

	ΔG_top-pose_	ΔG_ensemble_	ΔG_ensemble_−T ΔS_CR_
RMSE	2.97	3.86	3.77
MAE	2.56	3.38	3.43
SD	1.4	2.0	1.6

**Table 4:** Energies of association (E_a_) of GFN2-xTB and DFT calculations for top-ranked CB[8]*G0–**G1** ligand-receptor complexes in kcal/mol.

CB[8]* Ligand	GFN2-xTB	PBE0	B2PLYP	PWPB95
**G0**	−38.61	−31.12	−37.68	−33.77
**G1**	−28.39	−19.57	−21.68	−19.66
**G2**	−30.34	−18.63	−23.20	−20.30
**G3**	−35.28	−22.31	−27.55	−24.42
**G4**	−35.79	−19.12	−24.51	−21.44
**G5**	−31.26	−22.57	−28.41	−25.15
**G6**	−28.31	−25.57	−29.70	−28.59
**G7**	−28.69	−11.53	−15.94	−12.96
**G8**	−22.46	−20.86	−21.69	−19.87
**G9**	−24.51	−22.37	−23.54	−21.44

## Data Availability

The data presented in this study are openly available as Supplementary Materials.

## Supplementary Materials

The following are available online at https://www.mdpi.com/1422-0067/22/6/3078/s1.
