# Supplementary material for: Accurate Receptor-Ligand Binding Free Energies from Fast QM Conformational Chemical Space Sampling
Source: Int J Mol Sci. 2021 Mar 17;22(6):3078. doi: 10.3390/ijms22063078 (PMC8002627; doi:10.3390/ijms22063078)
Supplement: Supplementary file 1 [file ijms-22-03078-s001.pdf]

Supplementary Information

Accurate Receptor-Ligand Binding Free Energies from Fast QM Conformational Chemical Space Sampling

Esra Boz [1], Matthias Stein[1,*]

[1] Max Planck Institute for Dynamics of Complex Technical Systems, Molecular Simulations and Design Group, Sandtorstrasse 1, 39106 Magdeburg, Germany; boz@mpi-magdeburg.mpg.de; matthias.stein@mpi-magdeburg.mpg.de

* Correspondence: matthias.stein@mpi-magdeburg.mpg.de

Table S1:

Table S1. Typical CREST settings

T /K		298.15	
Constrain force constant		0.5	
Wall potential:		ellipsoidal, polynomial	
list of V_{bias} parameters applied		V_{bias} prefactor k	V_{bias} exponent alpha
	Metadyn 1	0.00100	1.000
	Metadyn 2	0.00050	1.000
	Metadyn 3	0.00100	0.500
	Metadyn 4	0.00050	0.500
	Metadyn 5	0.00100	0.250
	Metadyn 6	0.00050	0.250

Table S2. Number of unique structures in the conformer-rotamer ensemble at each stage of the workflow (ligand-receptor complex/free ligand)

	**Number of Unique Structure in CRE of Complex/Ligand**		
**CB[8] Ligands**	CREST	Tight reoptimization	Hessian
**$G0^{2+}$**	16/12	10/12	8/12
**$G0^{+}$**	33/11	17/11	12/9
**G1**	63/11	22/11	21/11
**G2**	155/175	149/175	137/158
**G3**	4/5	4/5	4/5
**G4**	8/4	8/4	8/4
**G5**	10/9	10/9	10/9
**G6**	20/14	20/14	19/14
**G7**	43/17	43/17	39/16
**G8**	143/18	85/17	90/17
**G9**	206/23	193/23	175/22
